# Social Perception of Faces: Brain Imaging and Subjective Ratings

**DOI:** 10.3390/brainsci10110861

**Published:** 2020-11-16

**Authors:** Peter Walla, Minah Chang, Katrin Schaefer, Sonja Windhager

**Affiliations:** 1 CanBeLab, Department of Psychology, Webster Vienna Private University, Palais Wenkheim, Praterstrasse 23, 1020 Vienna, Austria; minah.chang@studenti.unitn.it; 2 School of Psychology, Centre for Translational Neuroscience and Mental Health Research, University of Newcastle, University Drive, Callaghan 2308, NSW, Australia; 3 Faculty of Psychology, Sigmund Freud University, Freudplatz 1, 1020 Vienna, Austria; 4 Faculty of Medicine, Sigmund Freud University, Freudplatz 3, 1020 Vienna, Austria; 5 Department of Evolutionary Anthropology, University of Vienna, Althanstrasse 14, 1090 Vienna, Austria; katrin.schaefer@univie.ac.at (K.S.); sonja.windhager@univie.ac.at (S.W.)

**Keywords:** facial adiposity, body fat percentage, social perception, EEG, ERP, N170, P200, N300, face processing, attractiveness, dominance, health, masculinity, maturity, social neuroscience

## Abstract

The aim of this study was to investigate how a female face is perceived in terms of its attractiveness, dominance, health, femininity-masculinity, and maturity in direct relation to the body fat percentage (BFP) conveyed by the face. To compare how young adults (ages 18 to 35) respond to different levels of body fat percentage both subjectively and objectively we collected survey ratings and electroencephalography (EEG) data across five different levels of BFP from 40 participants. We adapted the experimental design from a prior behavioral study and used calibrated and morphed female face images of five different BFP levels. The results of the survey are in consensus with the previous study and assessed to be a successful replication. From the EEG data, event-related potentials (ERPs) were extracted from one electrode location (right occipitotemporal brain region) known to be particularly sensitive to face-stimuli. We found statistically significant differences in the amplitudes of the P200 component (194 ms post stimulus onset) between the thickest face and all four other BFP conditions, and in the amplitudes of the N300 component (274 ms post stimulus onset) between the average face and three other BFP conditions. As expected, there were no significant differences among the N170 amplitudes of all five BFP conditions since this ERP component simply reflects the processing of faces in general. From these results, we can infer that holistic face encoding characterized by the N170 component in the right occipitotemporal area is followed by serial evaluative processes, whose categorical and qualitative matrix and spatiotemporal dynamics should be further explored in future studies, especially in relation to the social constructs that were focused on in this study.

## 1. Introduction

The hierarchical nature of visual processing has long been studied through behavioral measures (e.g., reaction time studies), lesion studies (e.g., split-brain, visual agnosia), single-cell recordings, and various brain imaging methods. Visual processing in the human brain is often investigated non-invasively with event-related potentials (ERPs) using electroencephalography (EEG) and event-related fields (ERFs) using magnetoencephalography (MEG) for their excellent spatiotemporal and time course mapping advantages. Before temporally sensitive brain imaging modalities became accessible with relative ease across the scientific community, the study of brain processes was reserved to clinical settings. Face processing was studied in prosopagnosia patients as the etiology of various forms of face recognition impairment were ascribed to the specific locality of focal lesions. The propensity for right-side biased lateralization in face processing was already noted in early clinical findings when face identification defects were observed in prosopagnosia patients with right, but not left, occipitotemporal lesions [1,2]. Using positron emission tomography (PET), Sergent et al. [3] could indeed confirm the occurrence of stronger right-lateralized activation of the ventro-medial occipitotemporal region in response to face recognition in normal subjects. Some have suggested that face processing in the left-hemisphere might be involved in low-level semblance that acts as a precursor for the right-hemisphere [4,5]. The left-right hemisphere dynamics in early face processing (i.e., before 170ms) is still inconclusive, but later occurring processes, such as face recognition, most likely depend on a bilateral network [6].

Kanwisher et al. [7] reported the findings of a specific area in the fusiform gyrus that was subsequently named the “fusiform face area” (FFA), in which the blood-oxygen-level-dependent (BOLD) signal was significantly stronger in response to face-related stimuli than object-related stimuli. Since then, the FFA’s key role in face perception and its functional specificity has gained further evidence through various behavioral, neuropsychological, and neurophysiological studies [8]. These studies consistently show that BOLD activations induced by face detection, as well as face recognition, are most consistent and robust in the lateral mid-fusiform gyrus (FFA), whereas face-related activations in the superior temporal sulcus are more likely associated with variant facial features such as gaze, expression, and lip movement [7,8,9].

Growing evidence supports the converging view that face processing takes place throughout a distributed neural network that forms the core system of face perception, which includes the lateral fusiform gyrus, superior temporal sulcus, and inferior occipital gyri [9]. This widely accepted core system model suggested by Haxby et al. views face perception as a hierarchical process in which the occipital face area (OFA) distinguishes facial features and provides feedback to other core regions. The OFA has been associated with early perception of facial features, which activates around 100 ms post stimulus onset [10,11,12]. Findings from a transcranial magnetic stimulation (TMS) study suggest that the early processing that occurs in OFA (between 60–100 ms post stimulus onset) is a necessary mechanism for accurate face identification [11]. In support of this notion, bilateral lesions of the OFA are found to impair higher-level face processing (e.g., identity, gender) even when the FFA is normally activated, possibly due to the compromised integrity of the network among the regions involved in face processing [13].

The relatively famous N170 ERP component as a face-selective neural marker (although it also responds to other inputs) has been confirmed in hundreds of ERP studies. The face-related N170 effect is consistently observed in the right lateral posterior or lateral occipitotemporal areas starting around 110 ms post stimulus onset with a peak negative deflection at around 160 to 170 ms [14,15,16,17,18,19]. The N170 component is now widely believed to reflect “activity in a neural mechanism involved in the early detection of structural features characterizing human faces” [14] (Bentin et al., 1996, p. 557). As such, the N170 component is found to be missing in prosopagnosia patients with a selective deficit in face recognition [20]. The hypothesis that the right-hemisphere dominant N170 for face-related stimuli is localized in the occipitotemporal sulcus [14] was initially confirmed in a functional magnetic resonance imaging (fMRI) study by Puce et al. [21], which found localized face-selective activation in the right occipitotemporal and inferior occipital sulci. Many later studies have since confirmed the theory by Bentin et al. [14]. For example, the dipolar source location of the face-related N170 and M170 (for MEG) in the fusiform gyrus was confirmed in a simultaneous neuroimaging study (EEG and MEG) by Deffke et al. [22], a strong correlational relationship between the face-related N170 component, the FFA, and superior temporal sulcus on the right side was confirmed in another simultaneous recording study (EEG and fMRI) by Sadeh et al. [23], and Barbeau et al. [6] tracked the N170 component in posterior and middle fusiform gyrus, and also in the lateral occipital cortex using intracerebral recordings.

In general, the face elicits an enhanced N170 amplitude compared to a non-face stimulus, and its peak amplitude presumably signifies brain activities in which a holistic face is distinguished from a non-holistic face or other non-face object [16,18,19,24,25].

Numerous studies have confirmed that faces are first processed as their perceptual whole rather than by their individual features [15,16,18] It also seems that the FFA is capable of carrying out the low-level function of holistic face encoding even without any input from the OFA [26]. As an initial piece of evidence, a simultaneous EEG–fMRI study [23] has shown that the activities of OFA are not highly correlated with the N170 component. More recently, Kadipasaoglu et al. [27] investigated the temporal dynamics of the OFA and the FFA using intracranial EEG and fMRI on nine patients about to go under neurosurgical procedures. They found no significant difference for the onset timing of face selectivity between the OFA and the FFA in the right hemisphere, and for the signal propagation latencies between the early visual cortex to the OFA and the early visual cortex to the FFA. The study also found that the feedforward connectivity from the early visual cortex to the FFA precedes bidirectional connectivity between the OFA and the FFA. Although inconclusive based solely on these results, Kadipasaoglu et al. [27] and others, e.g., [26], suggest that the processing of invariant face features might be better supported by a parallel network model than the traditional hierarchical model. The parallel network model theory is also supported by the stereo-EEG study by Barbeau et al. [6], which investigated the spatiotemporal dynamics of face recognition in 18 pre-surgical patients. The study found that there is an early establishment of the face processing network around 110 ms in the fusiform gyrus and inferior frontal gyrus, and overlapping parallel processes occurring at 240 ms and 360 ms along the ventral visual pathway. Importantly, the N170 component was seen to encompass later occurring processes at 240 ms in several different brain regions [6]. Therefore, our study focuses on both the N170 as well as the later occurring ERP effects at the same sites.

Ever since the discovery of the N170 component as the detector of human facial features, face processing research in the fields of cognitive sciences has made much progress, largely in thanks to various brain imaging modalities. However, the way we evaluate faces at later cognitive stages (e.g., qualitative evaluation) has been studied for a much longer time in social sciences among other research fields. From a theoretical point of view, the social perception of faces in humans is driven by evolutionary fitness principles wherein perceptual valence (i.e., positive and negative) is implicitly linked to qualities such as averageness, symmetry, sexual dimorphism, as well as overt cues of pathogen infection and overall health [28,29]. Relevant behavioral studies have found that people implicitly associate pathogen infection with obesity as a heuristic cue in automatic perceptual judgement [30] and that people use facial adiposity as a reliable cue in judgement of health [31]. As an important matter of methodological consideration for the studies on the social perception of faces, studies show that people can reliably estimate the overall adiposity of a person based on the perception of weight in the face, and that there is a robust correlation between facial adiposity and perceptions of health and attractiveness [31,32].

In the previous social perception paper that forms the basis for this study, Windhager et al. [33,34] created prototypes of calibrated and morphed images to isolate body fat percentage (BFP) as the independent variable of interest. They acquired 274 social perception ratings on five corresponding face images of different BFPs and found consistent response patterns across different age groups and sexes. Their study found that BFP is a reliable predictor for the perception of attractiveness, dominance, health, and masculinity, but not for maturity in young women [33,34]. If such qualitative judgements are in fact based upon biological underpinnings (e.g., evolutionary drives) rather than purely cognitive processes, we reasoned that the bottom-up effect of these specific evaluative neural processes could be discerned in neuroimaging. More specifically, the qualitative judgement represented by social perception ratings would be a consequence of multiple stages of neural processes encompassing input (i.e., a face stimulus), invariant feature encoding of the face (i.e., the N170 component), and automatic evaluation in the brain that leads to the behavior of conscious judgement.

The aim of this study was to replicate the behavioral study of Windhager et al. [34] using a larger number of images (100 images instead of five) and to incorporate an additional quantitative method (objective measurement) using EEG. We recorded brain activity changes while participants viewed the 100 images prior to obtaining the subjective ratings. We hypothesized that the behavioral data obtained would reflect a similar pattern as those found by Windhager et al. [34]. For the EEG data, we anticipated that there would be no significant difference for the N170 effect across the five BFP conditions at the right occipitotemporal electrode sites. However, we hypothesized that the BFP might influence the later occurring ERP effects at the same site.

## 2. Materials and Methods

### 2.1. Participants

A total of 40 young adults (20 females and 20 males between the ages of 18 and 35; international cohort from various different countries) took part in this study for a compensation of EUR 10. The mean age of the participants was 24 years (SD = 3.81). All participants reported being right-handed, having normal or corrected-to-normal vision, and not having any neuropathological history. The study was approved by the International Review Board (SU18-09) of Webster University (Saint Louis, MI, USA).

### 2.2. Visual Stimuli

The visual stimuli comprised of 100 calibrated and morphed female face images. Using twenty calibrated and morphed female face images of average BFP (22.7%) as the baseline, four more image sets were created by decrementing or incrementing the average BFP by minus 2 SD, minus 5 SD, plus 2 SD, and plus 5 SD of BFP along the geometric morphometric shape regression. More specifically, the facial images per condition shared the same shape configuration but showed hair, eye, and skin color variations according to the original data set of the study population. See Windhager et al. [33,34] for details on shape regression and morphing.

The EEG part of the study always took place before the survey in order to maintain perceptual novelty for the stimuli, which is an important consideration in brain imaging measures. The 100 images were presented in a random order with Psychology Software Tools E-Prime 2.0^®^ on a Dell E2214hb 21.5” widescreen LED monitor. The face images were presented as a neck-up overlay on a uniform grey background with a black screen frame. Each image was presented for one second, followed by a blank black screen (1 s), followed by a white fixation cross on a black background (1 s) and followed by another blank black screen (1 s) before the next stimulus onset.

In the survey part of the study, the same 100 images were presented in a random order with PsychoPy2 software [35] on a Dell P2317Hf 23” widescreen LED monitor. Each image was shown for five seconds on a black background, followed by a screen with five horizontal, analogue rating scales from 0 to 100 for the following social perception domains: attractiveness (hardly at all attractive–very attractive), dominance (submissive–dominant), health (unwell appearance–healthy appearance), femininity–masculinity (feminine–masculine), and maturity (child–adult). The opposite ends of the scales were labeled with their corresponding representational values, which were later converted into numeric values for statistical analysis. Each rating scale required the participant to drag an onscreen slider (default value = middle) to a desired point along the scale (0 to 100) and make a left click on the mouse once the decision was made. The next trial was initiated when all five ratings were completed. All responses were automatically saved on a scale from 0 to 100. After every 20 trials, the participants were given the option to take a short break. The average time it took to complete the survey part of the experiment was 35 min, not including the breaks.

### 2.3. Electroencephalography (EEG)

Electrical brain activity of each participant was acquired with a Geodesic EEG™ System 400 with the HydroCel Geodesic Sensor Net of 64 electrodes embedded with silver chloride sensors. The potential changes were continuously sampled at a rate of 1000 Hz with an EGI Net Amps 400 amplifier (Electrical Geodesics, Inc., Eugene, OR, USA) with a built-in Intel^®^ chip under an applied online low-pass filter of 50Hz. The continuous EEG data were recorded by EGI Net Station 5.4 software.

### 2.4. Procedure

The entire experiment was conducted in the CanBeLab (Cognitive and Affective Neuroscience and Behavior Lab) at the Webster Vienna Private University campus. Once the participants arrived at the lab, they were guided through a checklist to confirm that they met all inclusion criteria for the study. A written consent was obtained from all participants. They were then seated in a comfortable chair to have their head dimensions measured for the center point of the scalp (Cz point) and the correct size of the EEG net. After applying the EEG net over the whole scalp, the electrodes were connected to the ground, referenced to the Cz point and kept below 50 kΩ impedance. The participants were given instructions on how to stay still comfortably for the next 6 to 7 min while their EEG data were being recorded. They were also instructed to blink if needed only when they see a fixation cross on the screen between stimulus displays.

### 2.5. Data Analysis

**Behavioral data.** The subjective ratings for attractiveness, health, dominance, femininity–masculinity, and maturity across the five BFP conditions were averaged across each condition and across all participants. Descriptive analysis as well as correlation analysis were performed to show and compare relationships between BFP and social perception rating performance (see below).

**Physiological data.** The EEG signal processing and extraction were carried out with the EEGDISPLAY 6.4.9 software (Fulham, see acknowledgements). For each EEG data set, an offline bandpass filter from 0.1 to 30 Hz was applied before generating epochs from 100 ms before stimulus onset to stimulus offset (1 s presentation time). The duration of 100 ms prior to stimulus onset was used as the baseline. All epochs contaminated by visible artifacts were manually selected and excluded, and those with the electrooculogram (EOG) amplitude exceeding ±75 mV were automatically excluded. The ensemble average of each data set was re-referenced to the common average across all electrode sites. The cumulative ensemble average (Figure 2) was constructed from all 40 data sets. Finally, only data collected from one electrode location (right occipitotemporal; see electrode distribution insert in Figure 2) known to be particularly sensitive to face-related processing were further processed and calculated.

**Statistical analysis.** Behavioral data were descriptively analyzed, and Pearson’s correlations were calculated to test possible correlations between BFP and every single social perception rating performance. ERP amplitudes collected from the selected right occipitotemporal electrode location were reduced to three 16 ms long intervals (averages over 4 sample points) covering three distinct time points, 162 ms (referring to the well-known N170 ERP face component), 194 ms (referring to the P200 ERP component) and 274 ms (referring to the P300 ERP component), all showing maximum amplitudes of their respective ERP component. Single mean values were calculated for each 16 ms time window and with those repeated measures analyses of variance (ANOVA) were conducted within subjects and including all 15 conditions (5 “face conditions” ∗ 3 “time points”). Following that, for each of the three time points, the single mean amplitudes of every possible pair of face conditions were statistically compared across all 40 participants by calculating paired-sampled *t*-tests.

## 3. Results

### 3.1. Behavioral Data

The mean subjective ratings for each BFP condition across the five social perception domains are presented in Table 1.

**Figure 1:**
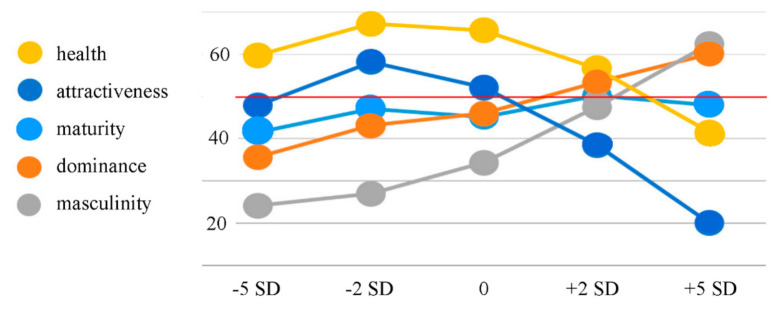
Averages of social perception rating data (N = 40; scale from 0 to 100; the mid-range value is marked with a red line). All five social perception categories are color-coded (see legends on the left). The x-axis shows the five face categories with increasing body fat from left to right. As in the previous study by Windhager et al. [34], the health curve and the attractiveness curve are in the shape of an asymmetric cap with an extreme deviation for the +5 *SD* BFP, the maturity curve is somewhat independent of the body fat percentage (BFP), and the dominance and femininity/masculinity curves are steadily rising.

The general pattern and shape of the curves in the following line graph (Figure 1) closely resemble those that were found in the previous study by Windhager et al. [34]. This is interpreted as a solid replication, which represents an important basis for this study since it is a follow-up investigation of Windhager et al.’s study [34].

Pearson’s correlations revealed a highly significant positive correlation between BFP and dominance ratings (*r* = 0.579; *p* = < 0.001), a significant positive correlation between BFP and maturity ratings (*r* = 0.171; *p* = 0.015), a highly significant positive correlation between BFP and masculinity ratings (*r* = 0.679; *p* < 0.001), a highly significant negative correlation between BFP and health ratings (*r* = −0.370; *p* < 0.001), and a highly significant negative correlation between BFP and attractiveness ratings (*r* = −0.575; *p* < 0.001). A false discovery rate correction calculation following the Benjamini–Hochberg procedure [36] revealed that no false positive results occurred.

### 3.2. Physiological Data

For the selected electrode location, and including all 15 conditions (five face categories and three time points), repeated measures ANOVA results revealed a non-significant Greenhouse–Geisser corrected overall “face condition” effect (*p* = 0.155; F = 1.741; partial Eta-square = 0.043). The factor “time” however resulted in a highly significant overall Greenhouse–Geisser corrected effect (*p* < 0.001; F = 28.267; partial Eta-square = 0.420) as well as the interaction of both factors “face condition * time” (*p* < 0.001; F = 4.619; partial Eta-square = 0.106). Follow-up *t*-tests revealed significant differences between the +5 *SD* BFP and all other conditions for the peak amplitudes of the P200 component at 194 ms post stimulus onset. Paired samples *t*-test revealed significant differences for the peak ERP amplitudes between +5 *SD* and −5 *SD* (*t*(37) = 3.565, *p* < 0.001), between +5 *SD* and −2 *SD* (*t*(37) = 3.705, *p* < 0.001), between +5 *SD* and the average (*t*(37) = 3.607, *p* < 0.001), and between +5 *SD* and +2 *SD* (*t*(37) = 2.427, *p* = 0.02).

**Figure 2:**
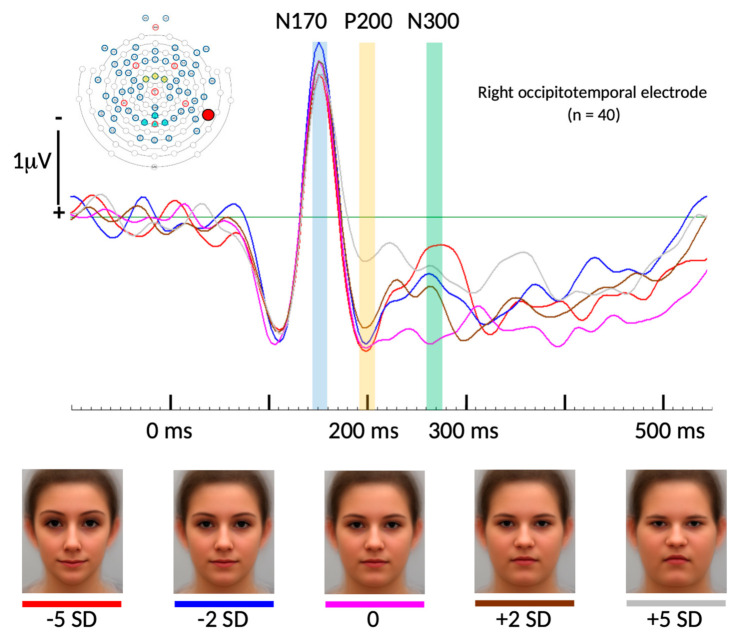
Event-related potentials (ERPs) of all five face conditions averaged across all 40 participants measured at one right occipitotemporal electrode location (the exact position is marked in the top left electrode distribution figure). At the bottom, all five face categories are shown with color codes matching their respective ERPs. No significant difference was found for the N170 component across the five BFP conditions (time point marked in blue color). For the P200 component, there were statistically significant differences between the peak amplitude of the +5 *SD* BFP condition and all others (time point marked in yellow color). For the N300 component, there were statistically significant differences between the peak amplitude of the average BFP condition compared to all others except for the +2 *SD* BFP condition (time point marked in green color).

**Figure 3:**
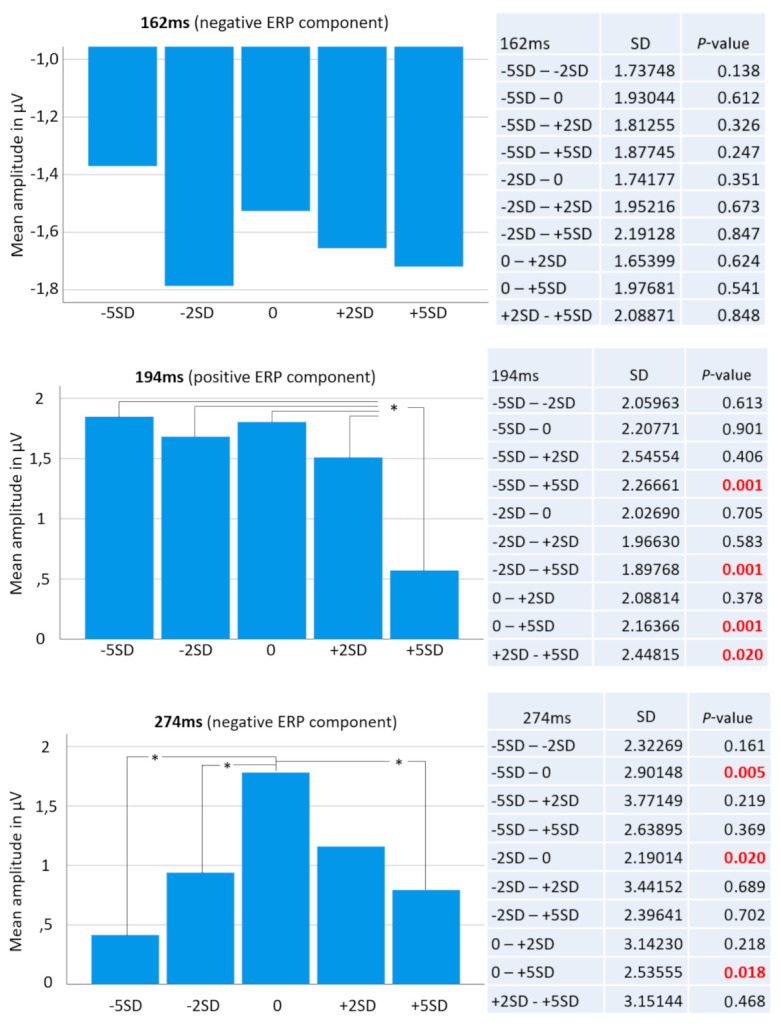
The top bar diagram shows mean ERP amplitudes at 162 ms post stimulus across all five face categories. Right o it is a table showing all possible pairs of mean amplitude comparisons including respective *t*-test results. No significant differences were found between any possible pairs of mean amplitude at this time point, which is known as the face-specific N170 ERP component. The middle bar diagram shows mean ERP amplitudes at 194 ms post stimulus across all five face categories. The table on the right shows all possible pairs of mean amplitude comparisons including respective *t*-test results. Strikingly, the +5SD face category (thickest face) was significantly different from all other categories. Finally, the bottom bar diagram shows mean ERP amplitudes at 274 ms post stimulus across all five face categories and right o it is a table showing all possible pairs of mean amplitude comparisons including respective *t*-test results. Here, the “normal” or “standard” face turned out to elicit significantly different brain activity from the −5SD, the −2SD and the +5SD face categories. Significant comparisons are marked with an asterisk (*) in the bar diagrams.

At the same electrode site, we found significant differences between the average BFP and three of the four other conditions for the peak amplitudes of the N300 component at 274ms post stimulus onset. Paired samples *t*-test revealed a significant difference for the peak ERP amplitudes between the average and −5 *SD* (*t*(37) = −2.98, *p* = 0.005), between the average and −2 *SD* (*t*(37) = −2.432, *p* = 0.02), and between the average and +5 *SD* (*t*(37) = 2.427, *p* = 0.02). See Figure 2 and Figure 3.

As predicted, no significance differences were found among the five BFPs for the N170 component. Figure 2 shows the cumulative ensemble average ERPs for each of the BFP conditions from the baseline (−100 ms duration before stimulus onset) up to 550 ms post stimulus onset and Figure 3 shows bar diagrams of all amplitude means including complete respective *t*-test tables.

## 4. Discussion

Our behavioral data show that BFP is a reliable predictor for the judgement of *attractiveness*, *dominance*, *health*, and *masculinity*, but not for *maturity*. The curvature patterns for the subjective ratings of all five domains are a perfect replication of the patterns found in the previous study by Windhager et al. [34], where the *attractiveness* and *health* curves follow an asymmetric cap-shaped pattern with an extreme deviation for the +5 *SD* BFP for younger adult raters, the *dominance* and *masculinity* curves follow a steadily rising pattern, and the *maturity* curve is somewhat independent of BFP. The previous study also noted that these curvature patterns are consistent across sexes and age groups (i.e., adolescents, younger adults, and older adults). Our results additionally show that the consistency of these patterns is preserved in a culturally heterogeneous study sample that comes from various parts of Europe, America, Asia, and Africa. The results of our correlation analysis also show that the decreases and increases (depending on the social perception construct) in ratings correlate significantly (mostly with high significance) with increases in BFP. The higher the BFP, the higher the dominance ratings, the higher the maturity ratings, the higher the masculinity ratings, the lower the health ratings and the lower the attractiveness ratings.

According to Little et al. [28], the consistent standard of beauty across individuals and cultures is “one of the best-documented and robust findings in facial attractiveness research since the 1970s” (p. 1639). There might be some adaptive individual differences in how a face is perceived, but these differences are still constrained by the frame of evolutionary theory [28]. If facial attractiveness is largely a judgement driven by biology, there would be a bottom-up complex that decides whether or not a face is attractive even in the absence of any top-down input. As a case point, an fMRI study by Chatterjee et al. [37] found an increased level of activation in the ventral occipital regions within and adjacent to the FFA and lateral occipital cortex in passive viewing of beautiful faces. The finding also supports the notion that there is no single specialized area in the brain for perceptual processes, but different brain regions involved in certain visual stimulus processing (i.e., functional specialization) serve as the basis of our perception [38].

Another interesting suggestion is that the judgement of beauty is driven by reward [39]. A study by Hahn and Perret [40] found that there was stronger activation in the dopaminergic motivational system for men when viewing attractive compared to unattractive female faces. The same study also found that seeing faces of the desired sex increases activation in the orbitofrontal cortex and mediodorsal thalamus [40]. The evolutionary view (i.e., mate selection) of face processing has been also supported by studies such as by Carbon et al. [41], whose findings suggest that gender information seems to be processed earlier than attractiveness. In considering such evidence in favor of the evolutionary and biological frameworks, it could be said that perceptual appraisal of faces occurs automatically beneath the surface level of our conscious awareness.

Our behavioral results support the previous findings that facial adiposity is highly correlated to attractiveness judgement [31,32] in that the highest two BFP conditions were perceived to be less attractive than the lower BFP conditions. The strong correlation between the curvature patterns for *attractiveness* and *health* also support the previous findings that there is a strong correlation between perceived attractiveness and health [31,32]. We also found a strong correlational relationship between the curvature patterns for *dominance* and *masculinity*. As Windhager et al. [34] found, there seems to be a positive correlation between BFP and perceived *dominance* and *masculinity*.

With respect to brain imaging, we also found a visible difference between the pattern of waveforms for the average face versus the two thinner faces and the two thicker faces starting at around 190 ms. These different processing patterns may reflect the overlapping parallel processes that have been found to occur in later stages at 240 ms and 360 ms (times measured in intracerebral recording) along the ventral visual pathway, starting with the N170 component that encompasses later occurring processes in several different brain regions [6]. The P200 component has been suggested to be valence specific as an orienting index for relevant stimuli whereas later occurring ERP components likely reflect cognitive and affective processes [42,43].

A concurrent TMS and fMRI study by Pitcher et al. [44] demonstrated that there are dissociable cortical pathways for processing static versus dynamic facial aspects, which supports the invariant versus variant face perception networks theory by Haxby et al. [9]. Compared to static cues (e.g., photos), using dynamic stimuli (e.g., 3D videos) in future studies could perhaps offer more ecologically valid and richer information considering that our perceptual systems evolved and were tuned to extract social information from moving faces and bodies [29]. Additionally, behavioral and neurobiological evidence shows that attractiveness judgement stems from not only aesthetically pleasing characteristics, but an integration of cues involving physical appearance, inter-personal engagement, and emotional expression [40].

As an implication of our findings and as suggested by other authors [32,33,34], future studies using face images should consider standardizing BFP across all images to reduce adiposity-related confounds. In studies of facial adiposity, using a control measure to set up a baseline of preferred BFP level for each participant could also be considered. We cannot rule out that distinctive small-scale facial features of different BFP image morphs may have influenced the behavioral and physiological responses in this study (cf. also Windhager et al., [45]), because neutral facial expressions also convey emotional meaning [46]. For example, the corners of the mouth are slightly downturned in the +5 *SD* BFP images (probably due to fatty pads or water retention), whereas they seem slightly raised in the −5 *SD* BFP images, which may have inadvertently elicited affective elements in the viewers [47,48,49]. The thicker facial morphs also feature smaller eyes and lower eyebrows, which could have potentially influenced the social perception of the face images we used in this study. Along these lines, Windhager et al. [45] showed that raters overweighed small-scale variation in face shape when judging the health status in comparison to the global shape patterns associated with body mass index in male faces. Altogether, with the use of calibrated geometric morphometric morphs (for the statistical advantages, see Windhager et al., [34] in brain imaging, we hope bridging expertise of diverse disciplines might ramify into models of neural processing patterns, which can then be systematically tested over a variety of physical predictors in social perception, stereotyping and stigmatization from faces and bodies. Societal relevance comes not only from our evolutionary biological roots, but also lies in the increasing amount of social media use.

As a future perspective, we suggest conducting further analysis that tests more accurate links between behavior, as in rating performance (perceptual decisions), and brain activation. This study showed that face-related brain activation patterns at 200 ms post stimulus show a different distribution than only 100 ms later at 300 ms post stimulus. Sometimes, brain activation changes correlate directly with rating performance, but other times they do not. Within only fractions of a second, brain activation patterns can follow different logics, which are not mirrored in the chosen constructs to make conscious decisions about.

## 5. Conclusions

We explored how female faces might be perceived differently in relation to the body fat percentage (BFP) they convey. We found that BFP is a reliable predictor for the social perception domains of *attractiveness*, *dominance*, *health*, and *masculinity*, and for *maturity* as it had been previously found. We also found that specific response patterns for social perception are preserved across a study sample composed of various cultural backgrounds. In our neurophysiological data, we found significant deviation in processing of the thickest face compared to all others, starting around 194 ms post stimulus onset at the right occipitotemporal area (i.e., the P200 ERP component). As hypothesized, the BFP did not influence the N170 component, only the later occurring brain potentials at the right occipitotemporal cortical area. We conclude that holistic face encoding characterized by the N170 component in the occipitotemporal area is followed by serial evaluative processes, whose categorical and qualitative matrix and spatiotemporal dynamics should be further explored in future studies.

## Figures and Tables

**Table 1:** The mean subjective ratings (*n* = 40 raters).

		Social Perception Domains				
		*Attractiveness*	*Dominance*	*Health*	*Feminity/Masculinity*	*Maturity*
BFP	−*5 SD*	48.04 (SD = 15.23)	35.76 (SD = 12.05)	59.79 (SD = 15.98)	24.22 (SD = 11.21)	41.63 (SD = 12.65)
	−*2 SD*	58.22 (SD = 12.52)	43.23 (SD = 8.71)	67.27 (SD = 14.21)	27.09 (SD = 8.85)	47.19 (SD = 10.98)
	*Average*	52.19 (SD = 12.72)	46.05 (SD = 7.52)	65.70 (SD = 12.80)	34.39 (SD = 12.75)	45.25 (SD = 11.77)
	*+2 SD*	38.62 (SD = 11.77)	53.40 (SD = 10.98)	56.73 (SD = 14.14)	47.57 (SD = 16.54)	50.25 (SD = 12.85)
	*+5 SD*	20.12 (SD = 13.55)	60.19 (SD = 17.33)	41.60 (SD = 19.72)	62.54 (SD = 20.28)	48.08 (SD = 15.31)
